# An Association between Decreased Small Intestinal RNA Modification and Disturbed Glucagon-like Peptide-1 Secretion under High-Fat Diet Stress

**DOI:** 10.3390/nu15173707

**Published:** 2023-08-24

**Authors:** Jiang Chen, Lin-Ling Deng, Xing-Lin Xiao, Shi-Yuan Long, Yuan Deng, Tong Peng, Jie Xie, Xiao-Yu Zhang

**Affiliations:** 1 College of Life Sciences, Sichuan Normal University, Chengdu 610101, China; chenjiang2019@163.com (J.C.);; 2 College of Life Sciences, Sichuan University, Chengdu 610065, China; pobo66@gmail.com; 3 Keystonecare Technology (Chengdu) Co., Ltd., No.200 Tianfu 5th Street, Chengdu 610094, China

**Keywords:** gut hormones, Western diet, microbiota, m6A, obesity

## Abstract

Unhealthy diets rich in fats and/or sugar are considered as the major external cause of the obesity epidemic, which is often accompanied by a significant decrease in gut hormone glucagon-like peptide-1 (GLP1) levels. Numerous studies have demonstrated notable contributions of the gut microbiota in this process. Nevertheless, the underlying mechanism still needs further investigation. The role of epigenetic modifications in gene expression and metabolism has been well demonstrated, with m6A methylation on RNAs being the most prevalent modification throughout their metabolism. In the present study, we found that the expressions of small intestinal *Gcg* and *Pc3*, two key genes regulating GLP1 expression, were significantly downregulated in obese mice, associated with reduced GLP1 level. Immunohistochemistry analysis indicated that a high-fat diet slightly increased the density of enteroendocrine L cells in the small intestine, implying that decreased GLP1 levels were not caused by the changes in L cell intensity. Instead, the small intestinal m6A level as well as the expression of known “writers”, *mettl3/14* and *wtap*, were found to be positively correlated with the expression of *Gcg* and *Pc3*. Fecal microbiota transplantation with feces from normal and obese mice daily to antibiotic-treated mice revealed that dysbiosis in diet-induced obesity was sufficient to reduce serum GLP1, small intestinal m6A level, and intestinal expressions of *Gcg*, *Pc3*, and writer genes (*mettl3/14*, *wtap*). However, as the most direct and universal methyl donor, the production of fecal S-adenosylmethionine was neither affected by the different dietary patterns nor their shaped microbiota. These results suggested that microbial modulation of the epitranscriptome may be involved in regulating GLP1 expression, and highlighted epitranscriptomic modifications as an additional level of interaction between diet and individual health.

## 1. Introduction

As a chronic multisystem disease, the prevalence of obesity (or excess adiposity) has increased in both developed and developing countries, contributing to 4 million deaths annually and 120 million disability-adjusted life-years [1]. Its healthcare cost was estimated at over USD 2 trillion worldwide [2]. Recent worldwide estimates suggest that almost 108 million children (~5% prevalence) and 604 million adults (~12% prevalence) are obese [1], and up to 58% of the adult population worldwide is predicted to be overweight or obese by 2030 [3]. In addition, accumulating evidence has indicated that obesity is closely related to the increased risk of non-alcoholic fatty liver disease, type 2 diabetes mellitus, dyslipidemia, premature aging, and atherosclerosis [4].

It is widely believed that unhealthy diets rich in fats and/or sugar are the major external cause of the recent obesity epidemic, aside from genetic factors [5,6]. Extensive studies have associated obesity with altered oxidative stress levels [7,8,9], a disturbed microbiota [10,11,12,13,14], and an abnormal endocrine system [15]. For example, the consumption of high-fat diets is capable of modifying the small intestinal morphology and pancreatic secretion, and attenuating the effects of fat on gastric emptying, gastrointestinal transit, antropyloroduodenal motility, as well as the secretion and action of gut hormones such as glucagon-like peptide1/2 (GLP1/2) [16,17]. Indeed, a fundamental relevance between GLP1/2 and the pathogenesis of obesity has been confirmed, making it a promising target for improving obesity [18,19].

GLP1 is produced from the post-translational processing of proglucagon (expressed by *Gcg*) by proprotein convertase 1/3 (PC1/3, encoded by *Pc3*) in L cells [16,20]. Well-established mechanisms underlying gut hormone secretion involve gut nutrient sensing by small intestinal enteroendocrine cells (EECs), through G-protein-coupled receptors (GPCRs), nutrient transporters, and ion channels [18]. Among them, GPCRs, as common sensory detection systems for many stimuli in EECs, mostly detect the small molecular products from nutrient digestion, such as free fatty acid receptors FFA1 and FFA4, monoacylglyceride receptor GPR119, bile acid receptor GPBAR1, and amino acid receptors CaSR and GPR142 [18]. In addition, microbiota-derived metabolites, such as SCFAs (signaling molecules for FFA2), deconjugated secondary bile acids (for GPBAR1), indole (for voltage-gated (K_V_) channels), and LPS (for TLR4), also modulate a range of EEC pathways like hormone biosynthesis and stimulus-secretion coupling pathways [18]. Taken together, both diet and its shaped microbiota could contribute to the gut hormones’ secretion, precisely the digestion products of nutrients and the metabolites of the microbiota. However, there is still a lack of insight on how these exogenous signaling molecules regulate hormone expression.

More recently, epigenetic regulation has attracted increasing attention, as a heritable mechanism altering gene expression and function without changes in the DNA sequence [21,22,23]. In addition to the well-established DNA methylation and histone modifications, reversible RNA modification has sparked a new wave of research in the field of epigenetics, with N^6^-methyladenosine (m6A) methylation on messenger RNA (mRNA) as the most prevalent modification in eukaryotes [24,25]. Numerous studies have shown that m6A is involved in the progression of diseases including cancer [26], cardiovascular diseases [27], as well as obesity [28]. m6A modification was conducted under the “writers” methyltransferase complex comprising core methyltransferase-like 3 (METTL3) and 14 (METTL14) subunits and other cofactors such as Wilms’-tumor-1-associating protein (WTAP) [29,30,31]. In particular, methyl donors provide methyl groups to finish the writing process, which are mainly involved in folate and the methionine cycle, such as folate, vitamin B6 and B12, methionine, choline, betaine, and S-adenosylmethionine (SAM) [32]. Among them, SAM acts as the most direct and universal methyl donor, whose synthesis depends mainly on the availability of others [33]. On the one hand, many of these donor substrates can be acquired from dietary components or nutrients, or generated from host-intrinsic pathways. From others, the microbiota is being increasingly appreciated as an additional source of these molecules, as well as that serve as cofactors or regulators of epigenetic enzyme activity [34,35,36,37]. For example, the microbiota belonging to probiotics *Bifdobacterium* spp. and *Lactobacillus* spp. is known to generate folate [38], and the commensal microbes can also metabolize dietary methionine into SAM [39]. SCFAs from the commensal microbiota showed an inhibition of deacetylase activity [40].

Taken together, intrinsic connections among diet, microbiota, and hormones showed a vital role in the onset and development of obesity, and might be associated with the microbiota-mediated m6A methylation, despite the fact that the mechanisms within it are still not fully investigated. Here, the decreased mRNA levels of *Gcg* and *Pc3* in the small intestine should be the direct cause of the reduced expression of GLP1, which showed a strongly positive correlation with changes in small intestinal m6A modification as well as the expression of “writers”. In particular, a dietary-shaped microbiota may act as a mediator to regulate the small intestinal m6A epitranscriptome. As far as we know, this is the first study revealing the relationship between gut hormone expression and microbiota-derived epigenetic modification, which highlights the microbiota-derived epitranscriptomic modifications as an additional level of interaction between diet and host.

## 2. Materials and Methods

### 2.1. Animal Studies and Procedures

All C57BL/6J mice were obtained from Chengdu DOSSY Experimental Animals Co., LTD (Chengdu, China). Animal experiments were approved by the Animal Ethics Committee at Sichuan Normal University (No. 2022LS028). Mice were adaptively kept under controlled conditions (20–22 °C at room temperature, 40–60% of humidity, and 12 h light/dark cycle by lights turning on at 8:00) with free access to sterilized food and water for 1 week. After that, mice were assigned to NC and DIO groups, and fed with sterilized normal-chew diet and high-fat diet (10% and 60% of calories derived from fat, respectively; see Supplementary Material Table S1), respectively, for 16 weeks.

For fecal microbiota transplantation (FMT), antibiotic-treated mice were firstly modeled by the orally given antibiotics mixture (20 mg/mL of Vancomycin, 40 mg/mL of metronidazole, 40 mg/mL of neomycin, and 40 mg/mL of ampicillin) once a day for 7 d, and then randomly assigned to FNC and FDIO groups, for the transplantation of feces from NC and DIO mice, respectively. Fecal suspensions were prepared by homogenizing daily collected feces with 0.9% sterile saline (1:10, *m/v*) and centrifuging at 800 g for 3 min. Both FNC and FDIO groups were firstly fed with the sterilized normal-chew diet mentioned above for 30 d and then sub-assigned to FNC-L and FNC-H, and FDIO-L and FDIO-H groups, with FNC-L and FDIO-L groups being continuously fed with normal-chew diet, while FNC-H and FDIO-H groups were fed with high-fat diet, respectively, for another 30 d.

General indicators such as body weight, blood GLP-1 level, fasting insulin, and fasting blood glucose, as well as biochemical and histopathological analysis, and fecal microbiota diversity analysis, were recorded or conducted according to our previous study [41]. Especially, feces for each group were collected for SAM quantification, while gastrointestinal tissues (the 1st, 2nd, 3rd, and 4th quarter of the small intestine, cecum, and the proximal and distal half of the colon) were simultaneously collected for further analysis, according to a previous study [42].

### 2.2. RT-qPCR Analysis

Segmented gastrointestinal tissues were homogenized with 1 mL of TRNzol solution. After that, one quarter of the segmented small intestine was mixed as the small intestine sample, while one half of the segmented colon was mixed as the colon sample. Total RNA of samples was firstly extracted with Tiangen TRNzol Universal Total RNA Extraction Kit according to manufacturer’s recommended protocol, with absorbance at 260 nm and 280 nm as indicators for the purity and yield of RNA. After that, RNA was then reversely transcribed into cDNA using FastKing Transcriptor First Strand cDNA Synthesis Kit, according to instructions. RT-qPCR analysis was performed using SuperReal PreMix Plus (SYBR Green) in TC-96/G/H(b)C PCR system (Bioer Biotechnology Co., Hangzhou, China), with the primers described in Supplementary Table S2, and *gapdh* was used as a housekeeping gene to calculate the relative gene expression using the 2^−ΔΔCt^ method.

### 2.3. Immunohistochemistry

To analyze the density of L cells, quadratic small intestines were stained with rabbit glucagon polyclonal antibody (15954-1-AP, Proteintech), and relevant secondary antibody IgG labeled with rhodamine (ZF-0316, ZSGB-Bio) with DAPI was used for cell localization. All immunofluorescent images were captured by a P250 FLASH fluorescence microscope (Danjier Electronics Co., Ltd., Jinan, China).

### 2.4. LC-MS Analysis for m5C and m6A Level

The total RNA acquired above was applied to determine the m5C and m6A levels. Briefly, 15 μg of extracted RNA was digested in 30 μL of the enzymatic buffer (25 mM Tris-HCl, 5 mM MgCl_2_, 0.5 mM ZnCl_2_, pH 9.0) at 37 °C for 1 h, with alkaline phosphatase (30 U) and snake venom phosphodiesterase I (0.08 U). After that, the digestate was added with 90 μL of ddH_2_O and extracted with 100 μL of chloroform three times, and the supernatants were combined for quantification of m5C and m6A levels.

Quantification was conducted on a UPLC PDA-QDa MS system under the method established by Chang et al. [43] and Yuan et al. [44], with small modifications. Briefly, 10 μL of samples was separated by a Waters AccQ-Tag^TM^ T3 column (1.7 μm, 2.1 × 100 mm) under 40 °C at 260 nm. Mobile phase A (0.1% formic acid in 10 mM ammonium formate aqueous solution) and B (Acetonitrile) were gradients at 0.25 mL/min as follows: 0–1 min, 97% A; 1–2.5 min, 97–90% A; 2.5–3.5 min, 90% A; 3.5–6.5 min, 90%-75% A; 6.5–6.51 min, 75–97% A; 6.51–11 min, 97% A. Measurement was performed using positive electrospray ionization (ESI^+^) mode with scan (m/z = 50~400) and selected ion record (SIR), to [M + 1]^+^ of the target subject (m/z of A = 268, m6A = 282, C = 244, m5C = 258). Solutions were infused from ESI source at 0.25 mL/min with the following parameters: capillary voltage 800 V, cone voltage 2V, drying gas 8 L/min, drying gas temperature 600 °C. Nitrogen was used as nebulizing and drying gas. All MS conditions were optimized to achieve maximal detection sensitivity. A typical chromatogram is shown in Supplementary Figure S1. Both concentrations of Adenosine (A), m6A, Cytidine (C), and m5C were acquired by external calibration using the standards, and was applied to calculate the m6A content using the following expressions:

m6A =×100

$$m5C\;\left(\%\right)=\frac{M_{m5C}}{M_{C}+M_{m5C}}\times 100$$

where *M_m_*_6*A*_ and *M_m_*_6*C*_ are the molar quantities of m6A and m5C, and *M_A_* and *M_C_* are those of A and C in the RNA samples, respectively.

### 2.5. ELISA for SAM Content Quantification

For quantitation of SAM content in feces and small intestinal tissues, collected samples were fully mixed with PBS buffer, and extracted by homogenization for 30 min under ice bath. After that, samples were centrifuged at 8000 g for 20 min, and the supernatants were applied for SAM quantification by commercially available enzyme-linked immunosorbent assay (ELISA) kits (Yinggong, Inc., Shanghai, China) according to manufacturer’s instructions.

### 2.6. Statistical Analysis

Statistical analysis was performed using SPSS 20.0 (Chicago, IL, USA), GraphPad Prism 5.01 (GraphPad Software, San Diego, CA, USA), and Origin Pro 9 (Origin Lab Corporation, Wellesley Hills, MA, USA). Significant differences were evaluated by one-way analysis of variance (ANOVA) and Tukey’s test under unpaired model. A value of *p* < 0.05 was considered statistically significant. All data are expressed as mean ± standard deviation (SD) with at least three duplicates.

## 3. Results

### 3.1. Decreased Expression of Gcg and Pc3 Is Consistent with the Lower GLP1 Level under High-Fat Diet

**Figure 1:**
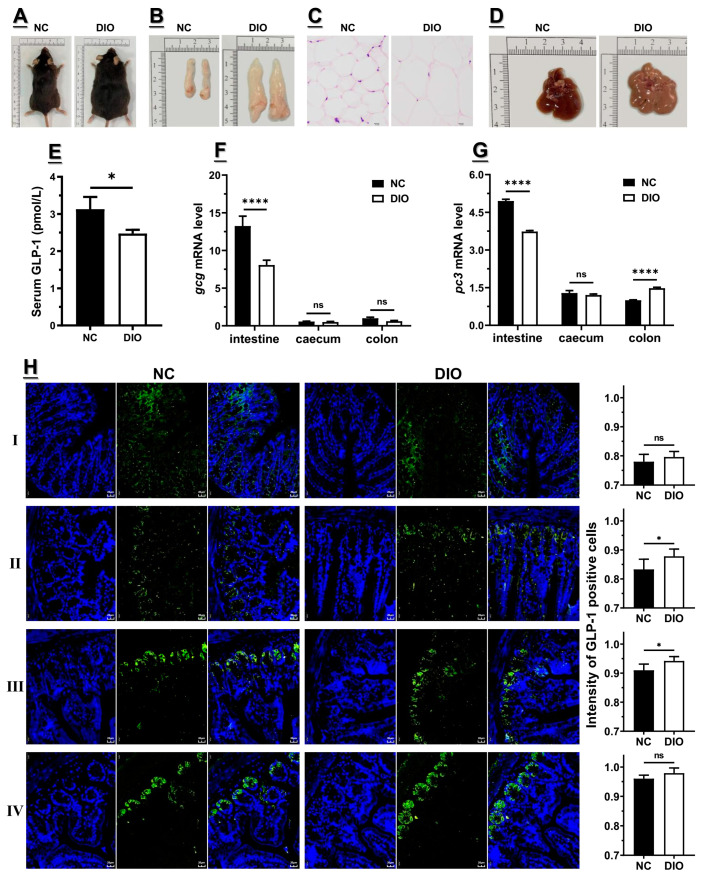
High-fat diet decreased GLP1 expression (n = 6). (**A**) Body habitus; (**B**) epididymis fat; (**C**) hematoxylin and eosin staining of epididymis fat; (**D**) liver; (**E**) blood content of GLP1; mRNA levels of (**F**) *Gcg* and (**G**) *Pc3* in different segments of gastrointestinal track; (**H**) representative immunofluorescent staining of GLP1-positive cells in the quadratic small intestines with DAPI (blue) and glucagon polyclonal antibody (green). For statistical differences, ns: *p* > 0.05, *: *p* < 0.05, and ****: *p* < 0.0001.

C57BL/6 J mice were fed with a high-fat diet for 16 weeks, resulting in a typical obesity feature compared with mice fed a normal-chew diet, including a significantly higher body weight and epididymal fat, as well as visible liver lesions (Figure 1A–D and Supplementary Figure S2A–D). Also, Lee’s index (*p* < 0.001), fasting blood glucose (FBG, *p* < 0.05), serum insulin (FSI, *p* < 0.05) levels, and the homeostasis model assessment-insulin resistance (HOMA-IR) index (*p* < 0.01) of DIO mice were significantly higher than those of NC mice, while the homeostasis model assessment-insulin sensitivity (HOMA-IS) index was exactly opposite (*p* < 0.001) (Supplementary Figure S1E–I). The total cholesterol (TC, *p* < 0.05), triglyceride (TG, *p* < 0.05), and high-density lipoprotein (LDL-C, *p* < 0.05) of DIO mice were also higher than those of NC mice, with a lower content of low-density lipoprotein (HDL-C, *p* < 0.05) (Supplementary Figure S1J–M). In addition, the blood GLP1 level of DIO mice was lower than that of NC mice (Figure 1E), consistent with the results from our previous study and others [17,41]. Both of the small intestine mRNA levels of *Gcg* and *Pc3* were consistent with blood GLP1 level, namely that the high-fat diet could downregulate the expression of *Gcg* and *Pc3* in the small intestine (*p* < 0.001) (Figure 1F–G), whereas the expression of *Gcg* and *Pc3* in the cecum appears to not be influenced by the high-fat diet. Instead, in the colon, the expression of *Gcg* was slightly downregulated under the high-fat diet (*p* > 0.05), while the expression of *pc3* was upregulated (*p* < 0.001) (Figure 1F–G). The reduced consistency implied that the expression of *Gcg* and *Pc3* in the small intestine contributes significantly to the reduced blood GLP1 level under the high-fat diet.

Morphological analysis of four small intestinal segments by using immunohistochemistry was conducted, and the results showed that the density of enteroendocrine L cells was slightly enhanced under the high-fat diet (Figure 1H), as indicated by others [45,46]. This suggested that the downregulation of *Gcg* and *Pc3* expression in the small intestine under the high-fat diet was not due to changes in L cell density. Notably, the density of L cells showed a gradual increase from the proximal to distal small intestine, as reported by others [47,48,49]. Overall, the expression of *Gcg* and *Pc3* in the small intestine dominated the *Gcg* and *Pc3* expression among the total gastrointestinal tract, and the downregulation of these genes under the high-fat diet coincided with the decrease in blood GLP1 levels, implying that the downregulation of small intestinal *Gcg* and *Pc3* makes major contributions to the decreased level of blood GLP1 under the high-fat diet.

### 3.2. Decreased m6A Level Might Contribute to the Lower Gcg and Pc3 Expression Levels

**Figure 2:**
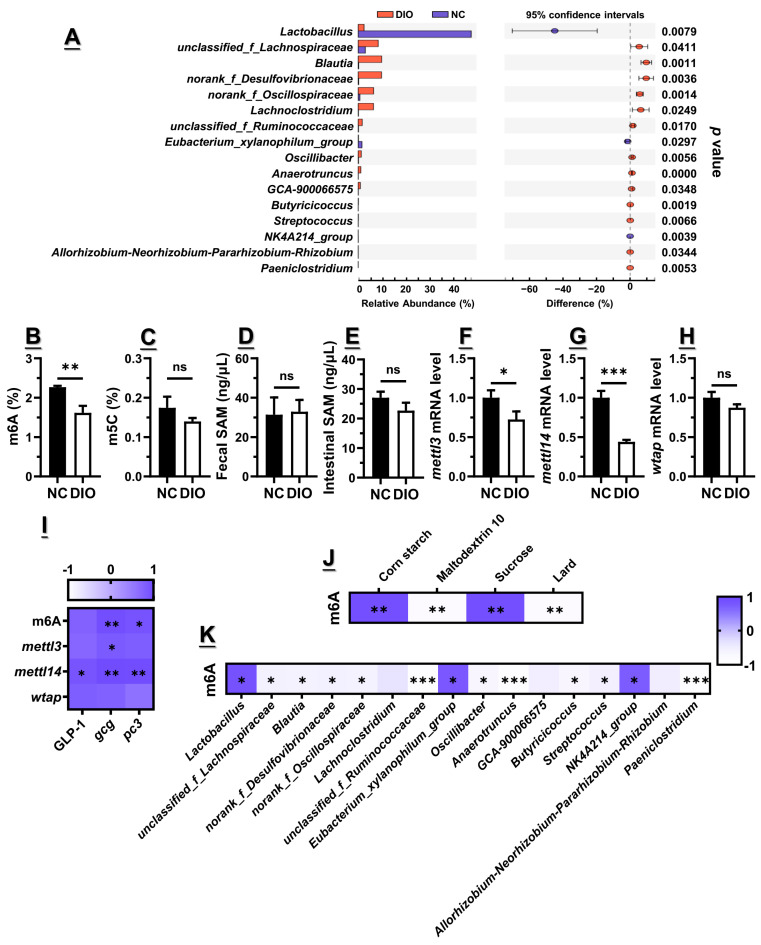
High-fat-diet-induced obesity was accompanied by abnormal GLP1 secretion, dysbiosis of microbiota, as well as decreased m6A methylation levels in small intestine (n = 6). (**A**) Differential bacteria on genus level, (**B**) m6A level, (**C**) m5C level, (**D**) fecal SMA level, (**E**) intestinal SMA level, as well as writers’ mRNA levels including (**F**) *mettl3*, (**G**) *mettl14,* and (**H**) *wtap* among NC and DIO mice. Correlation analysis of (**I**) GLP1 secretion and methylation, (**J**) m6A level and dietary components, and (**K**) m6A level and differential bacteria on genus level. For statistical differences, ns: *p* > 0.05, *: *p* < 0.05, **: *p* < 0.01, and ***: *p* < 0.001.

Analysis of methylation was focused on the small intestine, since the expression of *Gcg* and *Pc3* showed absolute predominance. As indicated, m6A (*p* < 0.01) and m5C (*p* > 0.05) levels of the small intestinal RNA from DIO mice were lower than those from NC mice (Figure 2B,C), but level of fecal SAM showed no difference (*p* > 0.05, Figure 2D), despite a slightly higher content of intestinal SAM for NC mice (*p* > 0.05, Figure 2E). This indicated that the decreased methylation level in the small intestine was not caused by the reduced availability of the universal methyl donor. Instead, expressions of “writers” including *mettl3* (*p* < 0.05), *mettl14* (*p* < 0.001), and *wtap* (*p* > 0.05) in the small intestine of DIO mice were downregulated (Figure 2F–H), both of which are involved in the formation of m6A on RNA. An apparently positive correlation between the m6A level of small intestinal RNA as well as the associated “writers” gene expression implied to us that the regulation of methylation modifications may be an alternative pathway through which diet regulates the expression of gut hormones (Figure 2I).

There are indeed differences in the composition of the used normal-chew and high-fat diets, as shown in Supplementary Table S1. Specifically, corn starch and sucrose rich in normal-chew diet showed significantly positive correlations with m6A level (*p* < 0.01 for both), while maltodextrin 10 and lard rich in high-fat diet were negatively correlated with it (*p* < 0.01 for both) (Figure 2J). However, as substrates that can be digested or metabolized by the microbiota, these components are unlikely to be directly involved in epigenetic modifications, which may require the microbiota to play a bridging role. As shown in Figure 2A, 16 genera of bacteria were found to be significantly differential between NC and DIO mice. In particular, *Lactobacillus* (*p* < 0.05), *Eubacterium_xylanophilum_group* (*p* < 0.05), and *NK4A214_group* (*p* < 0.05) showed significantly positive correlations with m6A level, where *unclassified_f_Lachnospiraceae* (*p* < 0.05), *Blautia* (*p* < 0.05), *norank_f_Desulfovibrionaceae* (*p* < 0.05), and *norank_f_Oscillospiraceae* (*p* < 0.05) were all negatively correlated with it (Figure 2K). Taken together, the above results suggested that decreased m6 methylation levels caused by the high-fat diet were derived from the reduced m6A epitranscriptome rather than the availability of methyl substrates, which may be originated from the disturbed microbiota.

### 3.3. Microbiota Dysbiosis Accompanied with Decreased GLP1, as Well as Gcg and Pc3 Expression Levels

After the 16-week consumption of the high-fat diet, the diversity of fecal microbiota from DIO mice showed a significant difference with that of NC mice, as indicated by the Shannon index (*p* < 0.05), despite no difference in Chao1 index between them (*p* > 0.05) (Supplementary Figure S3A,B). At the phylum level, the relative abundance of Firmicutes was significantly increased, while that of Bacteroidota was decreased (Supplementary Figure S3C). In particular, an extremely significant decrease in relative abundance of *Lactobacillus* at the genus level was observed (Supplementary Figure S3D). Principal component analysis (PCA) loading plots at the genus level further demonstrated the effect of the high-fat diet on microbial disturbance (Supplementary Figure S3E). The above results are generally consistent with the widely accepted consensus that the high-fat diet would disorder the fecal microbiota.

In addition, 16 genera of bacteria in total were found to be significantly differential between NC and DIO mice, with the top 10 as *Lactobacillus* (47.45 ± 15.64% for NC, and 2.45 ± 2.34% for DIO), *unclassified_f_Lachnospiraceae* (2.99 ± 2.12% for NC, and 8.39 ± 2.33% for DIO), *Blautia* (0.20 ± 0.09% for NC, and 9.84 ± 1.97% for DIO), *norank_f_Desulfovibrionaceae* (0.20 ± 0.09% for NC, and 9.79 ± 2.72% for DIO), *norank_f_Oscillospiraceae* (0.70 ± 0.39% for NC, and 6.47 ± 1.21% for DIO), *Lachnoclostridium* (0.09 ± 0.06% for NC, and 6.40 ± 3.12% for DIO), *unclassified_f_Ruminococcaceae* (0.11 ± 0.15% for NC, and 1.68 ± 0.68% for DIO), *Eubacterium_xylanophilum_group* (1.57 ± 0.76% for NC, and 0.10 ± 0.15% for DIO), *Oscillibacter* (0.12 ± 0.06% for NC, and 1.28 ± 0.37% for DIO), and *Anaerotruncus* (0.03 ± 0.02% for NC, and 1.10 ± 0.17% for DIO) (Figure 2A). Of interest was that only relative abundances of *Lactobacillus* and *Eubacterium_xylanophilum_group* were significantly reduced by the high-fat diet, while others among them were up-regulated.

### 3.4. Feces from DIO Mice Decreased Obesity-Associated GLP1 Level

**Figure 3:**
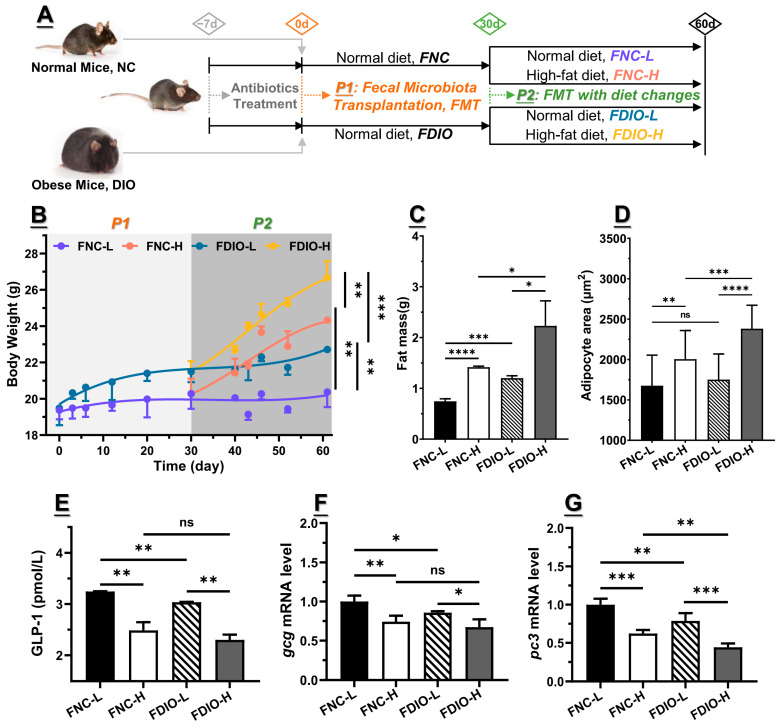
Obesity was intensified in antibiotic-treated mice receiving feces of DIO mice after being fed with high-fat diet (n = 6). (**A**) Procedure for fecal transplantation experiments: during the first 30 days (P1), antibiotic-treated mice were fed with sterilized normal-chew diet, with transplantation of feces from DIO and NC mice; next, high-fat diet was introduced for another 30 days (P2). (**B**) Body weight of mice was recorded at corresponding time (n = 6 for per group). At end, epididymis fat was collected for (**C**) weighting, and (**D**) adipocyte size measurement after H&E staining, as well as that of (**E**) blood GLP1, and (**F**) *Gcg* and (**G**) *Pc3* mRNA levels. For statistical differences, ns: *p* > 0.05, *: *p* < 0.05, **: *p* < 0.01, ***: *p* < 0.001, and ****: *p* < 0.0001.

To verify the above hypothesis, both feces of NC and DIO mice were transplanted to antibiotic-treated mice fed with a normal-chew diet for 30 days (P1, marked as FNC and FDIO, respectively), followed by changing the diet to a high-fat diet for another 30 days (P2, sub-marked as FNC-L and FNC-H, and FDIO-L and FDIO-H, respectively) (Figure 3A). As a result, increased body weight, accumulated epididymal fat, and enlarged adipocytes showed small differences among FNC-L and FDIO-L mice, as well as blood GLP1 level and *Gcg* and *Pc3* mRNA levels (Figure 3B–G, and Supplementary Figure S4, respectively), indicating that the effect of the microbiota shaped by the high-fat diet on developing obesity-related symptoms was somewhat limited. Specifically, during P2 when the normal-chew diet was changed to the high-fat diet, the difference between the above symptoms was increasingly intensified, such as the higher significant differences among body weight (Figure 3B), epididymal fat mass (Figure 3C), and lower *pc3* mRNA level (Figure 3G). Other indicators of obesity, including FBG, FSI, HOMA-IR, and HOMA-IS, as well as blood levels of TC, TG, LDL-C, and HDL-C, were also consistent with the above appearances (Table 1).

### 3.5. High-Fat Diet Aggravated Microbial Dysbiosis of Recipient Mice

**Figure 4:**
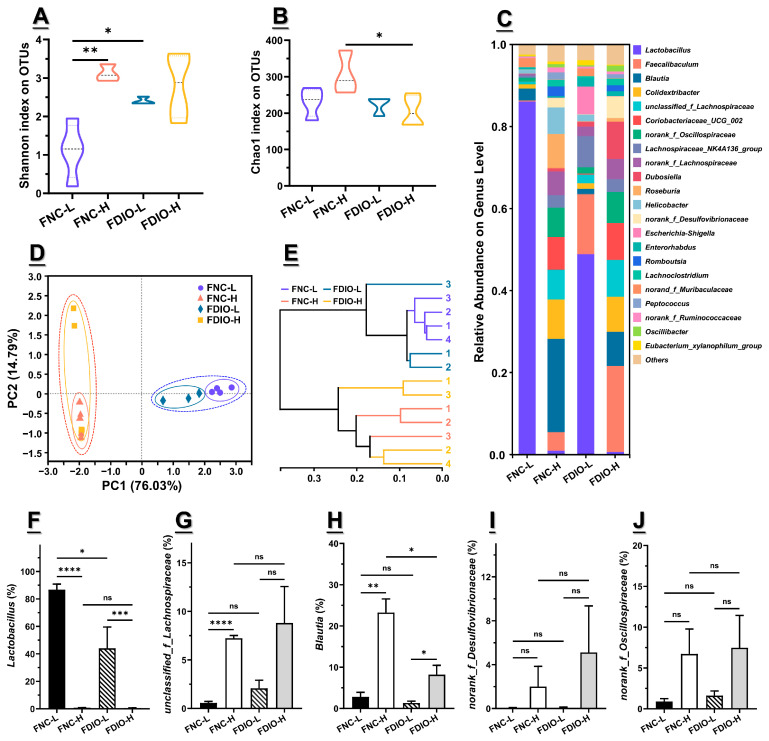
Microbial dysbiosis aggravated on antibiotic-treated mice receiving feces of DIO mice with high-fat diet. (**A**) Shannon index and (**B**) Chao 1 index of microbiota α-diversity on OTU level; (**C**) genus-level distribution of microbiota; (**D**) principal component analysis (PCA) score plot and (**E**) hierarchical clustering of fecal microbiota; (**F**–**J**) relative abundance of top 5 identified differential bacteria at genus level (n = 4 for FNC-L, n = 3 for FDIO-L, n = 3 for FNC-H, n = 4 for FDIO-H). For statistical differences, ns: *p* > 0.05, *: *p* < 0.05, **: *p* < 0.01, ***: *p* < 0.001, and ****: *p* < 0.0001.

Additionally, the microbiota diversity of recipient mice was analyzed. On an OTU level, Shannon index showed a major difference between FNC-L and the other three groups, and the Chao 1 index among them showed basically no difference, despite a significant one among FNC-H and FDIO-H (*p* < 0.05, Figure 4A,B, respectively), indicating that the microbiota of FNC-H, FDIO-L, or FDIO-H had a higher community diversity compared with FNC-L mice, which was directly or indirectly associated with the high-fat diet. This was supported by the results of the microbiota distribution on the genus level, in which the relative abundance of *Lactobacillus* in FNC-L mice accounted for more than 80%, largely reducing the community diversity of the microbiota, while the relative abundance of *Lactobacillus* was greatly reduced by the direct or indirect effect of the high-fat diet, enhancing the community diversity (Figure 4C). Further, PCA was conducted, with PC1 accounting for 76.03%, while PC2 accounted for 14.79% (Figure 4D). Distinct group-based clustering patterns were observed notably in the right quadrant composed of symbols representing FNC-L and FDIO-L mice (orange dotted circle), with the left quadrant composed of symbols representing FNC-H and FDIO-H mice (purple dotted circle), respectively, illustrating a drastic difference in fecal microbiota structure derived by diet patterns. Among them, subgroups could be further acquired based on the transplanted feces, with symbols representing mice transplanting feces from DIO mice mainly on the top quadrant (yellow and purple circles, respectively), and those receiving feces from NC mice mainly on the bottom quadrant (orange and blue circles, respectively) (Figure 4D). Hierarchical clustering analysis was consistent with the above results, as indicated by two diet-derived clusters and two feces-derived sub-clusters (Figure 4E). Taken together, dietary patterns appear to be the primary factor causing significant differences in the microbiota diversity of recipient mice, with the transplanted microbiota being the subsidiary factor.

Furthermore, the relative abundance of differential bacteria obtained from donor mice seems to be mainly regulated by diet than transplanted feces. In other words, changes in relative abundance of differential bacteria on recipient mice caused by fecal transplantation were further aggravated by diet. For example, the relative abundance of *Lactobacillus* was down-regulated from 86.72 ± 8.15% to 44.04 ± 31.05% by feces of DIO mice (*p* < 0.05), which was further decreased to 0.56 ± 0.63% after being fed the high-fat diet (*p* < 0.0001), resulting in a non-difference compared with mice receiving feces from NC mice and simultaneously being fed the high-fat diet (*p* > 0.05) (Figure 4F). Notably, the relative abundance of *Lactobacillus* was reduced to ~0%, when the normal-chew diet was changed to the high-fat one (*p* < 0.001). This is consistent with results from donor mice. On the contrary, the relative abundance of *norank_f_Desulfovibrionaceae* was firstly up-regulated from 0.57 ± 0.31% to 2.08 ± 1.66% by feces of DIO mice, and then increased to 8.80 ± 7.52% under the high-fat diet, despite no significant differences between them (*p* > 0.05) (Figure 4I). Data of the other top five differential bacteria obtained from donor mice showed similar trends (Figure 4G, H and J, respectively).

### 3.6. High-Fat Diet Exacerbated the Decreased m6A Level and Epitranscriptome

**Figure 5:**
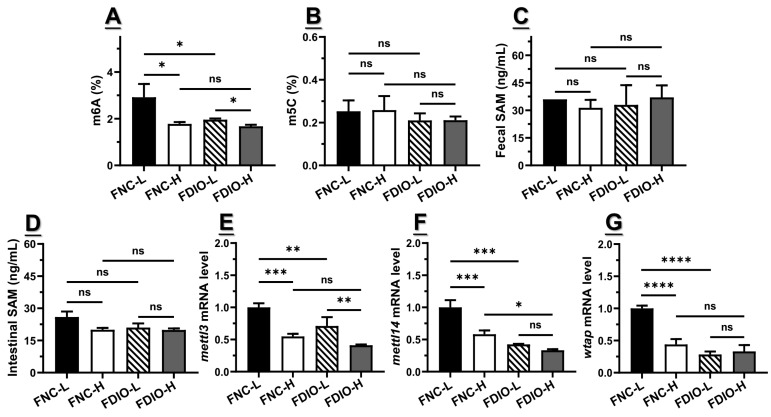
Methylation modification of antibiotic-treated mice fed with DIO feces was decreased by high-fat diet (n = 6). (**A**) m6A level, (**B**) m6C level, (**C**) fecal SAM level, (**D**) intestinal SAM level, as well as (**E**) *mettl3*, (**F**) *mettl14,* and (**G**) *wtap* expression levels of antibiotic-treated mice receiving DIO and NC feces fed with high-fat or normal-chew diet. For statistical differences, ns: *p* > 0.05, *: *p* < 0.05, **: *p* < 0.01, ***: *p* < 0.001, and ****: *p* < 0.0001.

Generally, m6A modification as well as its related epitranscriptome remained largely consistent with the distribution of obesity-related symptoms and differential bacteria mentioned above. For example, the m6A level of small intestinal total RNA in FDIO-L mice was significantly lower than that in FNC-L (*p* < 0.05), which was higher than that in FDIO-H (*p* > 0.05), indicating that the m6A level was firstly decreased by feces of DIO mice (from 2.92 ± 0.57% to 1.96 ± 0.05%), and was further decreased to 1.80 ± 0.23% by the high-fat diet (Figure 5A), while interestingly, there was still no significant difference in fecal SAM content on each group (*p* > 0.05, Figure 5C). Notably, the level of intestinal SAM for FNC-L mice was more or less higher than those of other groups (*p* > 0.05, Figure 5D). mRNA levels of *mettl3* (*p* < 0.01), *mettl14* (*p* < 0.05), and *fto* (*p* < 0.01) of FDIO-L mice were all also significantly decreased by the high-fat diet (Figure 5E–G). Under the high-fat diet, the m6A level and mRNA levels of *mettl3* and *wtap* of FNC-H mice showed no difference with those of FDIO-H mice, while mRNA levels of *mettl14* of FDIO-H mice were significantly lower than those of FNC-H mice. And the m5C levels of FNC-L and FNC-H were both higher than those of FDIO-L and FDIO-H (Figure 5B, *p* > 0.05). Taken together, the high-fat diet further reduced the obesity-related m6A level as well as the small intestinal epitranscriptome of mice receiving DIO feces.

In particular, the m6A level of FNC-L mice was significantly higher than that of FDIO-L mice (*p* < 0.05, Figure 5A), both of which consumed the normal-chew diet, which could theoretically promote methylation modification, by increasing the m6A and epitranscriptome expression levels. However, mice among these two groups received a different dietary-shaped microbiota, resulting in differences in their epitranscriptome, reflecting the important role of the microbiota in the dietary process involved in m6A methylation modification. In contrast, the epitranscriptome level of FNC-L mice was also significantly higher than that of FNC-H mice (*p* < 0.05, Figure 5A). Interestingly, both groups of mice received feces from NC mice but consumed different dietary patterns. This suggested that despite the importance of the normal-chew-diet-shaped microbiota, methylation modification was still largely inhibited due to the lack of an adequate diet, or to be precise, some dietary components like corn starch and sucrose. Taken together, the dietary-shaped microbiota may play as a mediator to regulate the induced epitranscriptome expression and subsequent epigenetic modifications, thus involving the expression of gut hormones such as GLP1 to contribute to obesity development.

## 4. Discussion

Given that the increasing incidence of obesity has been linked to the unhealthy eating habits characterized by high fat and/or high sugar, uncovering the mechanisms underlying the onset and development of diet-induced obesity has been going on for decades. As a digestive and endocrine organ, the gastrointestinal tract plays an important role in this process, partly due to the gut hormones and commensal microbiota [10,11,18,50]. Notably, microbiota-derived metabolites, such as SCFAs and LPS, have been linked to the gut hormones’ GLP1 secretion [18,41,51]. Considering the fact that dietary components could shape the microbiota [52], there appears to be a signaling flow linking the “diet-microbiota-hormone secretion”, despite the fact that digestion products of dietary components have long been shown to be the key signaling molecules to stimulate the expression and secretion of gut hormones [53]. Here, growth of *Lactobacillus* was inhibited to an extremely low level under a high-fat diet with a high content of maltodextrin and lard, accompanied with the downregulation of small intestinal *Gcg* and *Pc3* expression. This may be the direct cause of the decrease in GLP1 levels in obese mice. However, since GLP1 is produced from the post-translational processing of proglucagon by proprotein convertase, there is still a lack of deeper evidence to explain whether and/or how the microbiota regulate the expression of these genes.

Epigenetic modification has recently attracted more attention and has been increasingly recognized as another potent mechanism through which the microbiota showed its positive or negative effects on human health [25,35,36,37]. Here, we found that m6A and m5C levels of small intestinal total RNA were decreased by the high-fat diet, reaffirming the relationship between epigenetic modifications and diet-induced obesity [54]. In addition, the strong positive correlation between m6A and GLP1 level as well as *Gcg* and *Pc3* expression implies that RNA epigenetic modification may be involved in the expression of *Gcg* and *Pc3*, thus regulating GLP1 secretion. Extensive studies have found that epigenetic modification may be the hinge connecting microbiota and human health, mainly involving the following two accepted pathways: (1) the microbiota generate methyl donors to directly serve as epigenetic substrates for methylation modifications, for example, folate, VBs, and the universal SAM; (2) microbiota-derived metabolites such as the SCFAs mentioned above act as cofactors or regulators of epigenetic enzyme activity to indirectly be involved in methylation modifications [35,52,55].

However, it seems that none of our results can be reasonably explained by the above pathways. For one thing, a high-fat diet would decrease the production of fecal SCFAs [56], thus cutting down the possibility of regulating epigenetic enzyme activity. For another, fecal SAM levels here had no significant difference among NC and DIO mice, while intestinal SAM levels of NC mice were slightly higher than those of DIO mice. In addition, as a classical microbiota-derived by-product, LPS was also found to increase the GLP1 expression, especially in the distal intestine [51], which corresponded with the distribution of L cells in the gastrointestinal tract [47,48]. Nevertheless, this is unreasonable to account for the significant reduction in blood GLP1 levels, given that the high-fat diet would increase the production of LPS. Instead, we found that expressions of epigenetic “writers” were down-regulated under the high-fat diet, including METTL3/14 and WTAP, both of which are required for m6A deposition in cells [30]. Therefore, in addition to affecting the methyl donors’ SAM levels, downregulation of the above genes may play the main role in the decline in m6A levels. Actually, studies from Sabrina et al. have demonstrated that methyltransferase METTL16 would be downregulated in the absence of microbiota, resulting in less methylation of its target mRNAs for encoding SAM-synthase MAT2a [34]. Chen et al. found that *Fusobacterium nucleatum* could induce a dramatic decline in m6A modifications by the downregulation of METTL3, contributing to the induction of colorectal cancer aggressiveness [57]. Combined with our findings, it could be cautiously concluded that except for regulating the epigenetic modification process through inhibiting/promoting the epigenetic enzyme activity or influencing the SAM availability, a disordered microbiota could also affect the expression of epigenetic enzymes themselves, thus affecting the m6A epitranscriptome and subsequent epigenetic modifications. Notably, we found that the high-fat diet slightly increased the density of endocrine L cells in the small intestine, consistent with previous results [45,46]. This might contradict the decreased blood GLP1 levels under a high-fat diet, and emphasize that the reduction in blood GLP1 levels under a high-fat diet may be more directly related to the level of gene expression rather than by affecting the number of enteroendocrine L cells.

As far as we know, this is the first study to link the secretion of gut hormones with epigenetic modifications. Despite that, small sample size could be one of the limitations of this study. In addition, the location of m6A and m5C modification is worth further exploration, as their levels of total RNA rather than their profile on specific RNA were acquired using the LC-MS method. For example, a mapping method is usually applied to reveal the fundamental features of transcripts containing an m6A modification as well as the potential mechanisms by which certain sites are selected for methylation [30,34,58], despite its disadvantages such as low conversion rate, misreading, long detection time, and complex chemical reaction [43]. In contrast, LC-MS is regarded as a standardized method for accurate measurement of m6A levels [58], under the premise of ignoring the dynamic change in m6A modification. Instead, we tend to believe that technologies for target RNA purification from others before LC-MS analysis would be innovated to improve its accuracy, which may help us to determine the locations where m6A modification occurs and reveal its physiological effects. Meanwhile, the present study was also limited by the in vitro examination on GLP-1-positive enteroendocrine cells, whose execution should rely on the isolation, identification, and acquisition of key metabolites and/or key gut microbes. Notably, we suggested that high-fat-diet-induced obesity is caused by abnormal epigenetic modifications from the diet-shaping microbiota, except for the diet itself. As described, methyl donors can be supplied with a diet, and these dietary-originated components can directly act as substrates for epigenetic modification [33,58]. However, contents of such substrates are equal in the used normal-chew and high-fat diet, among which differential components, including corn starch, maltodextrin 10, sucrose, and lard, cannot directly be utilized for methylation modification. This suggests again that the interaction between dietary components and the microbiota should be paid attention to. On the one hand, it has been widely accepted that dietary components participate in shaping the microbiota. Conversely, the microbiota could metabolize (or convert) dietary components into various metabolites, such as SCFAs and serotonin [10,11,50,56]. Therefore, in addition to searching for active metabolites, tracing their metabolic fluxes may help to fully reveal the relationship between diet and host health mediated under the microbiota.

In conclusion, despite the lack of the above processes, the strongly positive correlation between the m6A epitranscriptome and mRNA levels of *Gcg* and *Pc3* also suggested that epigenetic m6A modifications may play an important role in the secretion of gut hormones. Indeed, the effect of diet on the overall GLP1 level deserves further attention, and RNA methylation modifications such as m6A and m5C levels could be indicators to reveal the epigenetic events associated with GLP1 secretion, which can be used to evaluate the effects of dietary supplements, lifestyle, and dietary structure on metabolic syndrome. In-depth research on this area is needed to further reveal the mechanisms between diet and obesity development as well as the role of the microbiota among it, and to provide a therapeutic direction.

## Figures and Tables

**Table 1:** High-fat diet intensified obesity in antibiotic-treated mice receiving DIO feces (n = 6).

	FNC-L	FNC-H	FDIO-L	FDIO-H
FBG (mM)	4.40 ± 0.40	5.57 ± 0.12 **	5.3 ± 0.44	6.60 ± 0.40 ^#§^
FSI (mIU/L)	1.28 ± 0.07	1.47 ± 0.01 **	1.45 ± 0.06 ^&^	1.67 ± 0.06 ^##§§^
HOMA-IR	0.25 ± 0.01	0.32 ± 0.02 **	0.32 ± 0.02 ^&&^	0.42 ± 0.03 ^#§§^
HOMA-IS	0.18 ± 0.01	0.14 ± 0.01 **	0.14 ± 0.01 ^&&^	0.11 ± 0.01 ^#§^
TC (mM)	2.10 ± 0.14	3.13 ± 0.65 *	2.38 ± 0.11 ^&^	3.76 ± 0.51 ^##^
TG (mM)	0.45 ± 0.03	0.69 ± 0.03 ***	0.67 ± 0.08 ^&&^	0.74 ± 0.09
LDL-C (mM)	0.18 ± 0.02	0.24 ± 0.04	0.20 ± 0.03	0.28 ± 0.06
HDL-C (mM)	3.18 ± 0.27	1.92 ± 0.05 ****	2.43 ± 0.38 ^&^	1.65 ± 0.22 ^#^

Notes: FNC-L vs. FNC-H, * *p* < 0.05, ** *p* < 0.01, *** *p* < 0.001, **** *p* < 0.0001; FDIO-L vs. FDIO-H, ^#^ *p* < 0.05, ^##^ *p* < 0.01; FNC-L vs. FDIO-L, ^&^ *p* < 0.05, ^&&^ *p* < 0.01; FNC-H vs. FDIO-H, ^§^ *p* < 0.05, ^§§^ *p* < 0.01.

## Data Availability

The datasets used and/or analyzed during the current study are available from the corresponding author on reasonable request.

## Supplementary Materials

The following supporting information can be downloaded at: https://www.mdpi.com/article/10.3390/nu15173707/s1, Table S1: Ingredients of normal-chew and high-fat diet; Table S2: Sequence of the used primers; Figure S1: Typical mass chromatogram of A, m6A, C and m5C; Figure S2: High-fat diet induced a typical obesity on mice; Figure S3: High-fat diet induced the microbiota dysbiosis; Figure S4: Obesity was intensified on pseudo-sterile mice receiving feces of DIO mice after fed with high-fat diet.
