# Supplementary material for: An Association between Decreased Small Intestinal RNA Modification and Disturbed Glucagon-like Peptide-1 Secretion under High-Fat Diet Stress
Source: Nutrients. 2023 Aug 24;15(17):3707. doi: 10.3390/nu15173707 (PMC10490556; doi:10.3390/nu15173707)

Supplementary Materials

for

An Association between Decreased Small Intestinal RNA Modification and Disturbed Glucagon-like Peptide-1 Secretion under High-Fat Diet Stress

Jiang Chen [1,2,†], Lin-Ling Deng [1,†], Xing-Lin Xiao [1,†], Shi-Yuan Long [1], Yuan Deng [1], Tong Peng [2,3], Jie Xie [1] and Xiao-Yu Zhang [1,*]

1 College of Life Sciences, Sichuan Normal University, Chengdu 610101, China; chenjiang2019@163.com (J.C.)
2 College of Life Sciences, Sichuan University, Chengdu 610065, China; pobo66@gmail.com
3 Keystonecare Technology (Chengdu) Co., Ltd., No.200 Tianfu 5th Street, Chengdu 610094, China
* Correspondence: zhangxy2005@126.com
† These authors contributed equally to this work.

Content:

Table S1 Ingredients of normal-chew and high-fat diet

Table S2 Sequence of the used primers

Figure S1 Typical mass chromatogram of A and m6A

Figure S2 High-fat diet induced a typical obesity on mice

Figure S3 High-fat diet induced the microbiota dysbiosis

Figure S4 Obesity was intensified on pseudo-sterile mice receiving feces of DIO mice after fed with high-fat diet

Table S1 Ingredients of normal-chew and high-fat diet

Serial number	Normal-chew Diet (D12450B)		High-fat Diet (D12492)	
Macronutrient	**g%**	**Kcal%**	**g%**	**Kcal%**
Protein	19.2	20.0	26.2	20.0
Carbohydrate	67.3	70.0	26.3	20.1
Fat	4.3	10.0	34.9	59.9
Total	-	100.0	-	100.0
Kcal/g*	3.85		5.24	

Ingredient	**g**	**Kcal**	**g**	**Kcal**
Casein, 80 mesh meshes	200	800	200	800
DL-Methionine	3	12	3	12
Corn starch	315	1260	0	0
Maltodextrin 10	35	140	125	500
Sucrose	350	1400	68.8	275.2
Cellulose BW20	50	0	50	0
Soybean oil	25	225	25	225
Lard	20	180	245	2205
Complex mineral	10	0	10	0
Dicalcium phosphate	13	0	13	0
Calcium carbonate	5.5	0	5.5	0
Potassium citrate, monohydrate	16.5	0	16.5	0
Multivitamin V10001	10	40	10	40
Choline Bitartrate	2	0	2	0
FD&C Yellow Dye #5 Pigment	0.05	0	0	0
FD&C Red Dye #40 Pigment	0	0	0	0
FD&C Blue Dye #1	0	0	0.05	0
Total	**1055.05**	**4057**	**773.85**	**4057**

* Nutrient to calorie ratio (Kcal/g) was used to measure the amount of a specific nutrient (or group of nutrients) in a diet per calorie.

Table S2 Sequence of the used primers

Gene name	Primers
gcg	Forward: AGCGACTACAGCAAATAC
	Reverse: GTTCCTCTTGGTGTTCAT
pc3	Forward: AAGTGGTCGTTACAGATGTGA
	Reverse: AGAATCCTTGGTCCGTGTT
mettl3	Forward: ATCCAGGCCCATAAGAAACAG
	Reverse: CTATCACTACGGAAGGTTGGG
mettl14	Forward: CTGAGAGTGCGGATAGCATTG
	Reverse: GAGCAGATGTATCATAGGAAGCC
wtap	Forward: GCCCCAACGTTTAAGTGCAG
	Reverse: AGCATTCGACACTTCGCCAT
gapdh	Forward: TTCCAGTATGACTCTACCCACGGCA
	Reverse: GCACCAGCATCACCCCATTTG

Figure S1 Typical mass chromatogram of Adenosine, m6A, Cytidine and m5C.

(A) Total ion chromatography (TIC) of the digested samples; (B-E) Selected ion recording (SIR) of A, m6A, C, m5C.

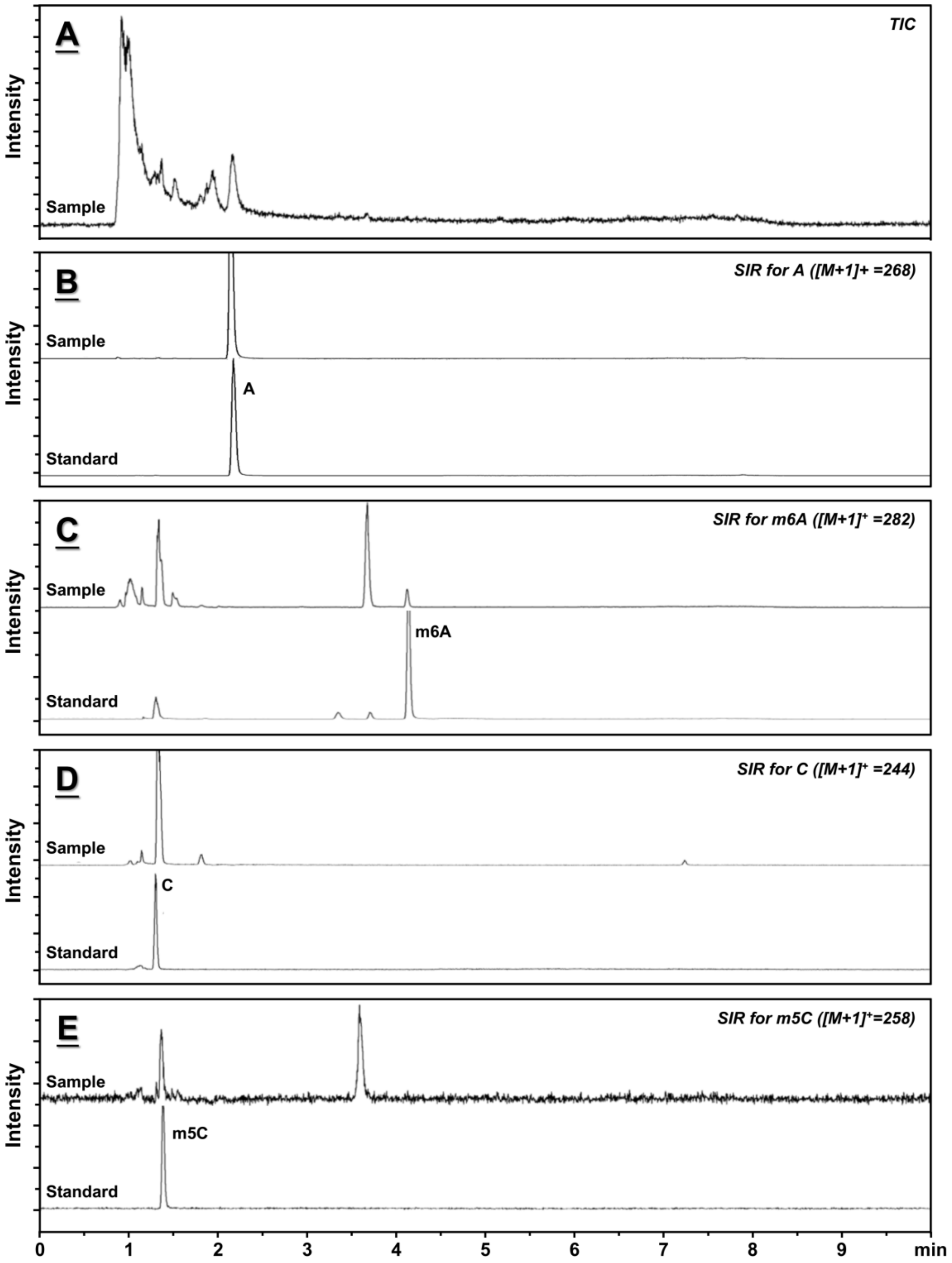

Figure S2 High-fat diet induced a typical obesity on mice (n =6).

(A) body weight; (B) fat mass; (C) adipocyte size of epididymis fat; (D) liver mass; (E) Lee's index; (F) fasting blood glucose; (G) fasting serum insulin; (H) homeostasis model assessment (HOMA)-insulin resistance (IR) index; (I) HOMA-insulin sensitivity (IS) index; Blood contents of (J) total cholesterol (TC), (K) triglycerides (TG), (L) low density lipoprotein (LDL) and (M) high density lipoprotein (HDL). For statistical differences, * for P<0.05, while ** for P<0.01, *** for P<0.001 and **** for P<0.0001.

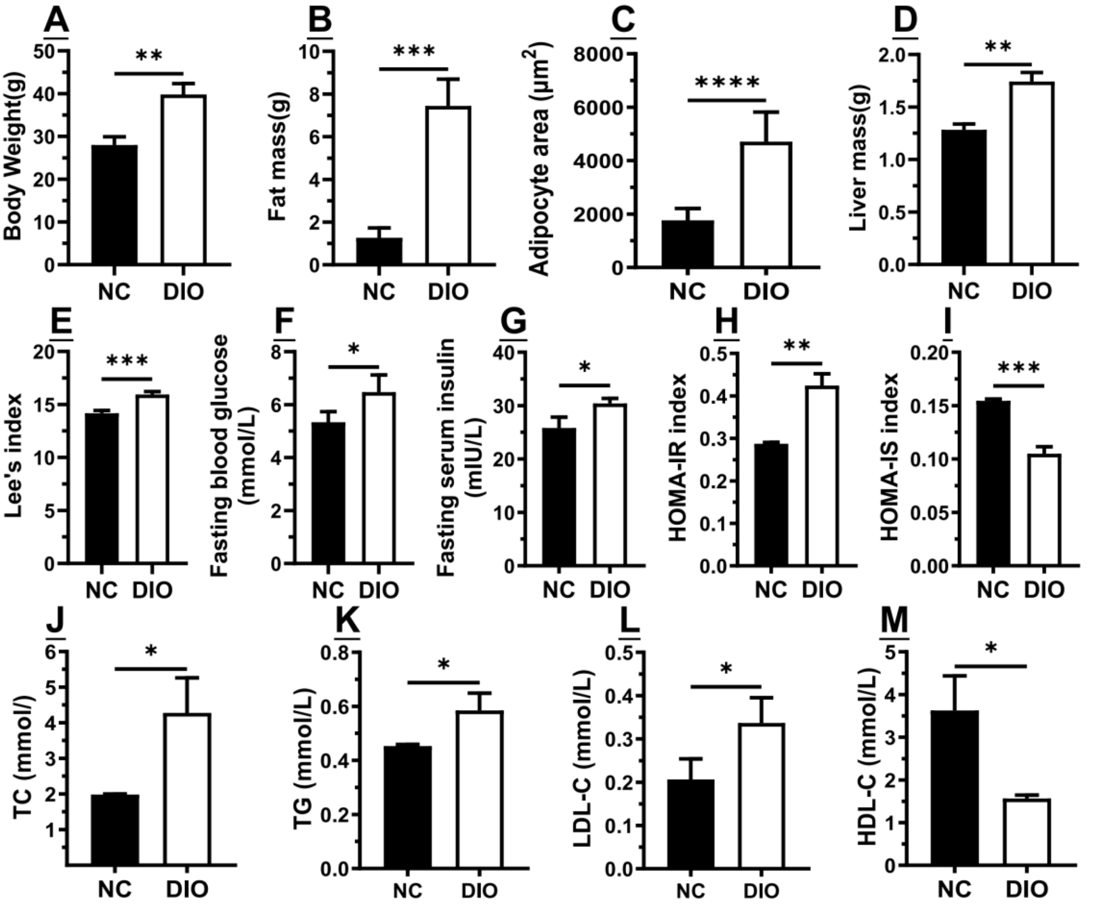

Figure S3 High-fat diet induced the microbiota dysbiosis (n = 3).

(A) Shannon index and (B) Chao1 index on OUT level of fecal microbiota of NC and DIO mice; (C) Phylum-level and (D) Genus-level distribution of fecal microbiota of NC and DIO mice; (E) Principal components analysis (PCA) of fecal microbiota. For statistical differences, ns indicates P>0.05, while * for P<0.05.

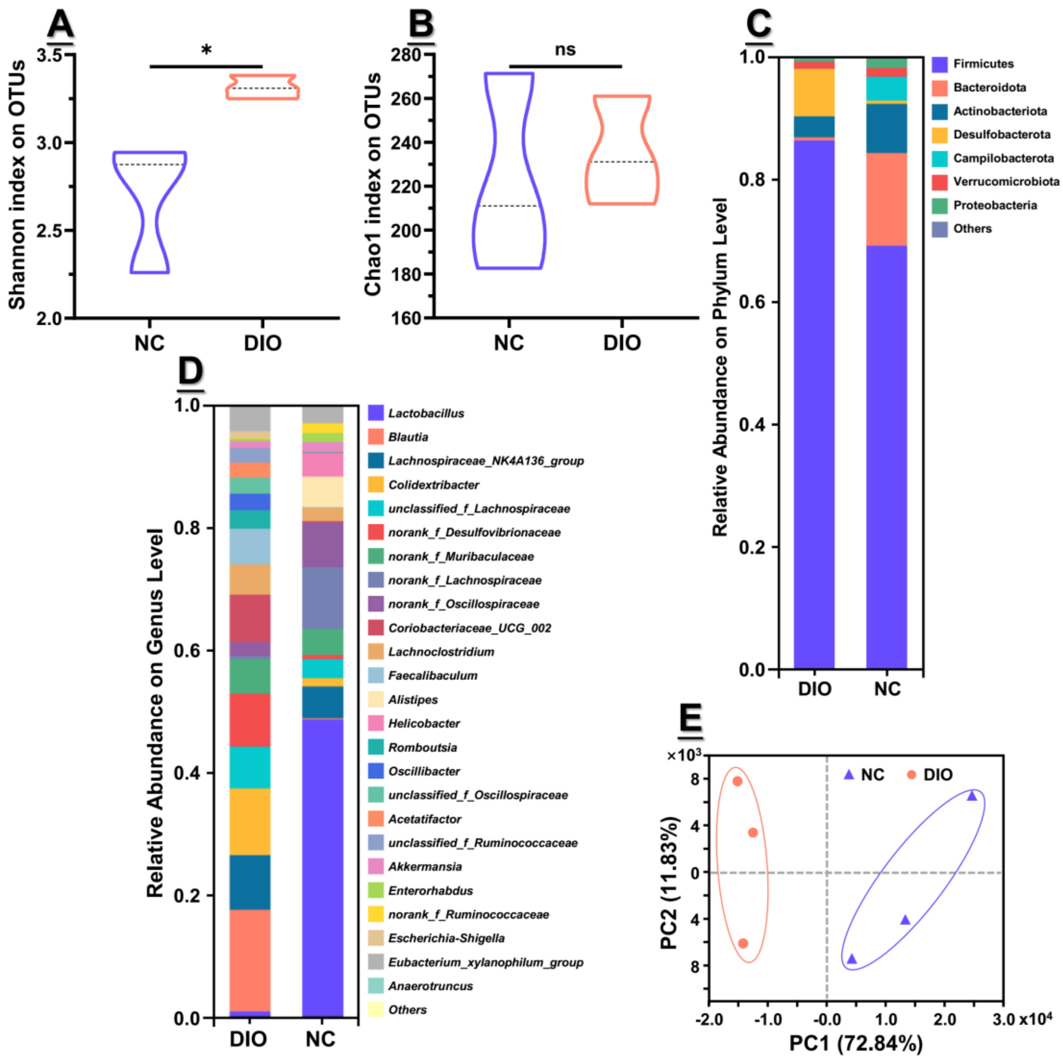

Figure S4 Obesity was intensified on pseudo-sterile mice receiving feces of DIO mice after fed with high-fat diet.

(A) habitus of mice and (B) H&E staining of their epididymis fat.

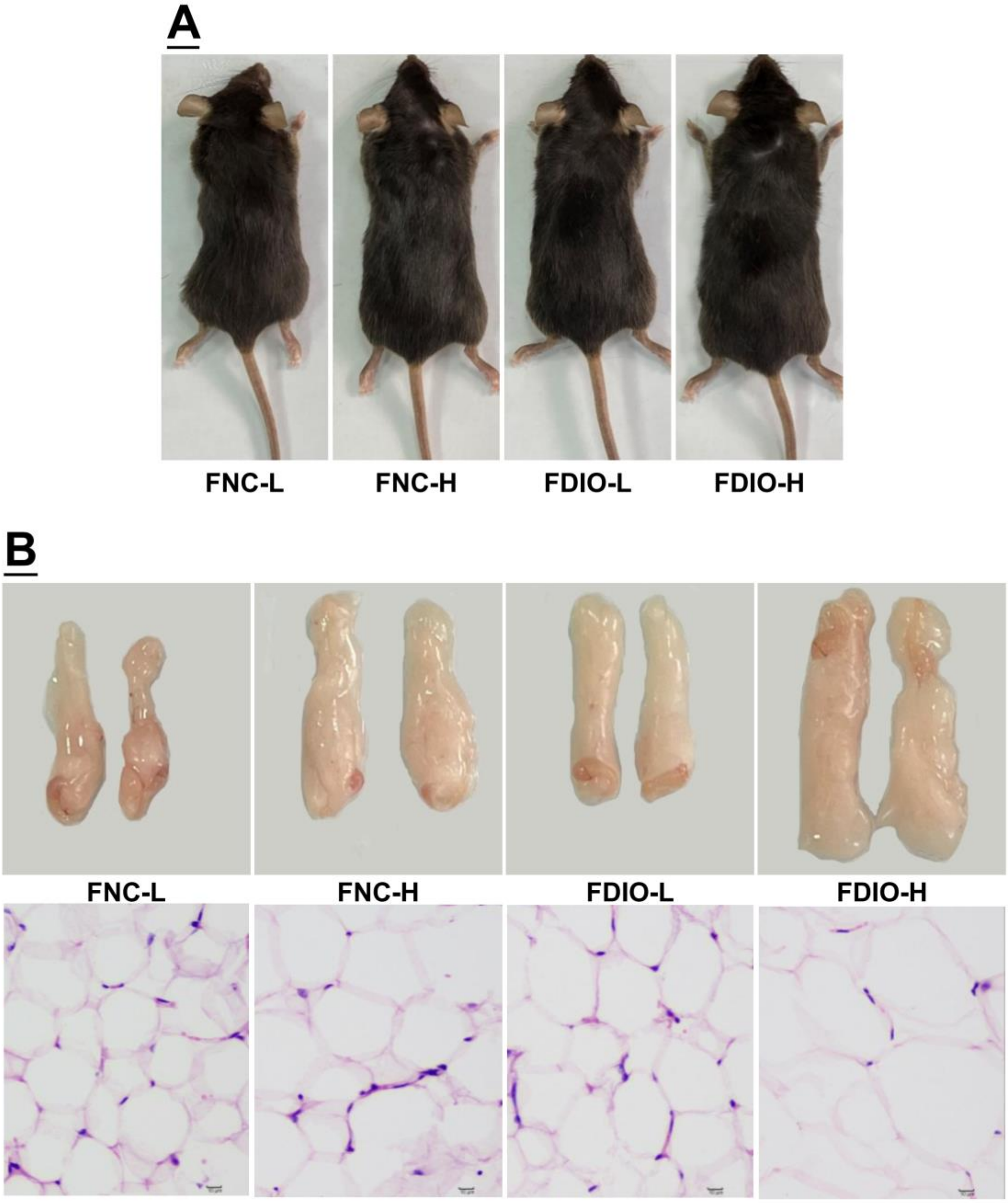

Supplement: Supplementary file 1 [file nutrients-15-03707-s001.zip › nutrients-2548131-supplementary.pdf]
